# Retrospective Evaluation of a Dexamethasone Sparing Antiemetic Regimen: An Antiemetic Prophylaxis Study on NEPA (Netupitant Plus Palonosetron) for Preventing Chemotherapy-Induced Nausea and Vomiting (CINV) in Cancer Patients

**DOI:** 10.7759/cureus.49763

**Published:** 2023-11-30

**Authors:** Suhas Agre, Madhura Agre, Pooja Pol, Mubarakunnisa Tonse, Mitasha Mohanty, Alfiya Shaikh

**Affiliations:** 1 Department of Medical Oncology, Cancer One Clinic, Mumbai, IND; 2 Department of Medical Oncology, Hinduja Hospital, Mumbai, IND; 3 Department of Medical Oncology, Cumballa Hill Hospital, Mumbai, IND

**Keywords:** emesis, 5-ht3ras, netupitant plus palonosetron (nepa), dexamethasone, chemotherapy-induced nausea and vomiting (cinv)

## Abstract

Background

Corticosteroids, specifically dexamethasone (DEX), have been extensively utilized for the prevention of chemotherapy-induced nausea and vomiting (CINV). However, their usage is associated with a range of adverse events. In contrast, the combination of Netupitant Plus Palonosetron (NEPA) with a single dose of DEX provides comparable efficacy in preventing CINV over a five-day period following chemotherapy administration. This regimen offers the advantage of reducing the need for additional doses of DEX, particularly in the high-risk setting of HEC (Highly emetic chemotherapy).

Objective

To evaluate dexamethasone sparing anti-emetic regimen (single dose dexamethasone with NEPA) for prophylaxis of CINV in patients receiving HEC.

Methodology

This is a retrospective, observational, real-world, single-center study including data of 69 patients who received high‐dose emetogenic chemotherapy and were administered DEX (8 or 12 mg) on day 1, with no dose of DEX on days 2, 3, and 4, combined with an oral combination of tablet netupitant 300 mg and palonosetron 0.5 mg. NEPA was taken orally an hour prior to the start of the HEC cycle. The primary efficacy endpoint was complete response (CR) which is defined as no nausea, emesis, or no rescue medication during the Acute (< 24 hours) and Delayed Phase (25-120 hours) of chemotherapy.

Results

The overall CR achieved in the acute and delayed phase for vomiting is 100% at all four follow-ups. The CR achieved in the acute phase is 95.7% whereas 98.6% of patients showed CR in the delayed phase respectively. No patient required any rescue medication. No acute and delayed phase of vomiting was reported.

Conclusion

A simplified regimen of NEPA plus single‐dose DEX offers effective CINV prevention throughout five days post‐chemotherapy with the advantage of sparing patients additional doses of DEX in the high-emetic‐risk setting chemotherapy.

## Introduction

Chemotherapy-induced nausea and vomiting (CINV) is a distressing side effect commonly experienced by cancer patients undergoing chemotherapy. It not only compromises their quality of life but also affects treatment adherence and outcomes [1,2]. Incidence of CINV has been reported in as high as 60%-90% of chemotherapy-treated patients, globally [1]. The incidence of nausea tends to be higher than that of vomiting, and antiemetic medications tend to be less effective in controlling nausea, specifically in highly emetogenic chemotherapy. Anticipatory nausea appears to occur in approximately 29% of patients receiving chemotherapy (about one of three patients), while anticipatory vomiting appears to occur in 11% of patients (about one of ten patients) [3].

Highly emetogenic chemotherapy (HEC) such as Carboplatin, Carmustine, Cisplatin, Ifosfamide, Cyclophosphamide, etc. is commonly used in the treatment of metastatic cancers and is associated with a high risk of severe nausea and vomiting [4]. Traditional antiemetic therapies, such as 5-HT3 receptor antagonists (5-HT3RAs) and corticosteroids, have been effective in managing acute CINV [1]. However, delayed CINV, which occurs beyond 24 hours after chemotherapy administration, has proven to be more challenging to control. CINV is prevented by the administration of dexamethasone (DEX) in low dose of range 4mg-12mg. Cancer patients usually receive oral dexamethasone before the start of chemotherapy followed for up to four days post each chemotherapy infusion. DEX dampens the effect of CINV, however, it may also worsen the treatment-related insomnia, stress and fatigue, negatively affecting the treatment adherence [5]. DEX may inhibit CINV but it also suppresses the immune system [6]. To alleviate the debilitating effects, antiemetic regimens have been continuously refined and upgraded. One such regimen is the combination of NEPA (Netupitant plus Palonosetron), a dual-action antiemetic, specifically designed to target both acute and delayed phases of CINV. NEPA has shown promising results in preventing CINV, particularly in patients receiving high emetogenic chemotherapy (HEC) [2].

NEPA combines two distinct pharmacological agents, netupitant and palonosetron, each targeting different pathways involved in CINV. Netupitant is a neurokinin-1 (NK-1) receptor antagonist, which blocks the substance P receptor in the central nervous system, preventing the signal transmission that leads to nausea and vomiting [7]. Palonosetron, a second-generation 5-HT3RA, acts by inhibiting the serotonin receptors in the gut and brain, further alleviating the symptoms of CINV [8,9]. The improved efficacy of NEPA compared to traditional antiemetics is especially notable in the prevention of delayed CINV [10]. The extended duration of action provided by NEPA enables better control of symptoms in the delayed phase, reducing the need for rescue medications and enhancing patients' overall well-being during the critical post-chemotherapy period [11].

NEPA represents a significant advancement in the management of CINV in patients receiving HEC. Its unique dual-action mechanism, targeting both acute and delayed phases of CINV, provides comprehensive symptom control and improves patients' quality of life [12]. Although consistent efficacy and favourable safety profile of NEPA across various cancer types and chemotherapy regimens make it a valuable treatment option for clinicians. This study stems from the need to evaluate the role of NEPA in the management of CINV, particularly in patients receiving chemotherapy known to induce high emetogenicity.

## Materials and methods

Study & participants

This retrospective, single-center, open-label, single-arm, investigator-initiated study analysed the data of 69 patients diagnosed with HEC types of cancer conducted at the Cancer Cure Center from August 2022 to February 2023.

Ethical committee

The study obtained approval from the Institutional Ethics Committee.

Patients & selection

Key Inclusion Criteria

The study included adult patients of both genders aged 18 years and above who underwent HEC according to the National Comprehensive Cancer Network (NCCN) 2023 list of drugs. All participants had a confirmed diagnosis of cancer established through histological or cytological examination. Additionally, patients were required to have an Eastern Cooperative Oncology Group (ECOG) performance status ranging from 0 to 2, indicating their ability to perform daily activities with minimal limitations and exhibit acceptable hepatic function with transaminase levels not exceeding two times the upper limit of normal, as well as renal function with creatinine levels below 1.5 times the upper limit of normal.

Key Exclusion Criteria

The study excluded individuals who were less than 18 years old, pregnant or lactating women, those who had experienced a myocardial infarction within the last six months; individuals with a documented or known hypersensitivity to 5HT3RA (5-Hydroxytryptamine Receptor 3 Antagonists) or NK1RA (Neurokinin-1 Receptor Antagonists) and their excipients; patients with uncontrolled diabetes mellitus; patients who reported nausea and vomiting at baseline; and those with gastrointestinal obstruction or active peptic ulcer.

Procedure

Dexamethasone in the dose of 8 or 12 mg was administered on day 1 only of each cycle. The patients when interviewed reported that the single dose of dexamethasone was combined with an oral combination of tablet netupitant 300 mg and tablet palonosetron 0.5 mg, 1 hour before the start of the HEC cycle.

Clinical objectives

The primary objective was to evaluate the complete response after any HEC administration and use of no rescue medication during the overall phase of all HEC cycles.

Secondary objective

The secondary objective was to evaluate the following:

A. Complete Response post-HEC in the acute phase (0-24 hours after chemotherapy (CT)) and delayed phase (25-120 hours).

B. Complete Control: By evaluating complete response with a maximum grade of mild nausea during the acute phase and delayed phase, along with no reported episodes of vomiting separately on the single days of all chemotherapy cycles.

C. Emesis-free duration: During the acute, delayed, and overall (0-120 hours) phase for each cycle of 21 days and separately on single days of all CT cycles, up to four cycles. Also, during the period between two consecutive cycles.

D. Nausea: During the acute, delayed, and overall phase of the 21-day cycle and separately on all days of chemotherapy cycles, up to four cycles. Also, during the period between two consecutive cycles.

E. Rescue medications required, if any.

Statistical analysis

The statistical analysis was done using SPSS version 25.0 (IBM Corp., Armonk, NY, USA). Basic descriptive analysis was done by calculating mean (± SD), percentage and frequency for various parameters included in the study.

## Results

Patients

The population of females (55.1%) and males (44.9%) was similar and the mean age of the study population was 51.93 years. Around 31.9% (n=22) of patients had hypertension and 20.3% (n=14) of patients had diabetes mellitus. The data of the current study also showed that 13% of patients had anticipatory CINV as well at baseline (Table 1). The description of patients’ demographics and baseline characteristics is illustrated in Table 1. Among the patients included in the study, the majority were of breast cancer (26.1%) and head and neck cancer (29%), receiving doxorubicin, cyclophosphamide, and paclitaxel for Breast cancer and cisplatin, fluorouracil, docetaxel with radiotherapy followed by weekly cisplatin for head and neck cancer (Table 2).

**Table 1:** Demographic profile of the study population

Variables		N = 69
Median age		51.93 years
Gender (n)	Female	31
	Male	38
Comorbidity, n (%)	Hypertension	22 (31.9%)
	Diabetes Mellitus	14 (20.3%)
Anticipatory CINV, n (%)		9 (13%)
Chemotherapy cycles received (n)	1	11
	2	13
	3	18
	4	16
	5	6
	6	3
	9	2

**Table 2:** Patients receiving HEC and MEC chemotherapy for different cancer indications HEC: Highly emetogenic chemotherapy; MEC: Moderately emetogenic chemotherapy

Indication	Frequency	Chemotherapy received	Frequency
Breast cancer	18	Doxorubicin + Cyclophosphamide + Paclitaxel	18
Biliary tract carcinoma (BTC)	6	Cisplatin + Gemcitabine	9
Head and Neck cancer	20	Cisplatin + fluorouracil + docetaxel + Radiotherapy followed by weekly cisplatin	20
Nodular lymphocyte-predominant Hodgkin lymphoma (NLPHL)	3	Doxorubicin + Bleomycin + Vinblastine + Dacarbazine	5
Gall bladder cancer	1	Paclitaxel + Carboplatin	3
Ovarian cancer	1	5-fluorouracil + Mitomycin	1
SCC anal	1	Bleomycin + Etoposide + Cisplatin	1
Seminoma	1	Pemetrexate + Carboplatin	4
Non-small cell lung cancer (NSCLC)	4	Cisplatin	1
Recurrent Endocervical Adenocarcinoma with lung metastasis	1	Docetaxel	1
Prostate cancer	1	BFM 2009 (Daunorubicin)	1
Acute lymphoblastic leukaemia (ALL)	1	Oxaliplatin + 5-Fluorouracil	3
Colorectal cancer (CRC)	1	Docetaxel + Cisplatin	2
Oesophageal cancer	1		
Thyroid cancer	1		
Gastric carcinoma	2		
Classic Hodgkin lymphoma (cHL)	2		
Cervical cancer	1		
Bladder cancer	2		
Adenocarcinoma of the anal canal	1		

**Figure 1:**
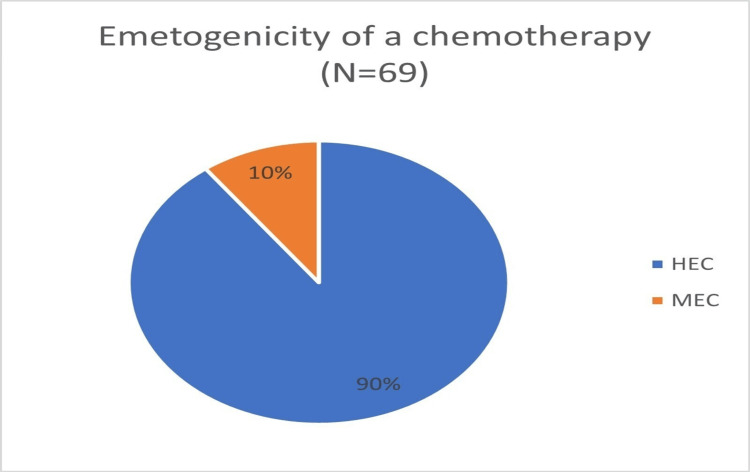
Emetogenicity of the chemotherapy study population received HEC: Highly emetogenic chemotherapy; MEC: Moderately emetogenic chemotherapy

Ninety percent of the population received highly emetogenic chemotherapy (HEC) and the rest received moderately emetogenic chemotherapy (MEC) (Figure 1).

Efficacy

Among 69 patients, 95.7% of patients showed a complete response for prophylaxis of acute CINV for the first cycle of chemotherapy and 98.6% of patients showed a complete response for prophylaxis of delayed CINV for the first cycle of chemotherapy. Post 4th cycle all patients had 100% CR rates for both acute and delayed CINV (Table 3).

**Table 3:** Complete Response (No Emesis, No Rescue Medication) Rates during Acute (<24 hours) and Delayed Phase (24-120 hours) with HEC and MEC CR: Complete response; CINV: Chemotherapy-induced nausea and vomiting; NEPA: Netupitant and palonosetron

Chemotherapy Cycles	Complete Response (CR)	Patients with No Emesis, No Rescue Medication required (N = 69)	CR (%)
Cycle 1 with NEPA	CR Acute CINV	66	95.7
	CR Delayed CINV	68	98.6
Cycle 2 with NEPA	CR Acute CINV	46	95.8
	CR Delayed CINV	46	95.8
Cycle 3 with NEPA	CR Acute CINV	35	100
	CR Delayed CINV	35	100
Cycle 4 with NEPA	CR Acute CINV	20	100
	CR Delayed CINV	20	100
Cycle 5 with NEPA	CR Acute CINV	5	100
	CR Delayed CINV	5	100
Cycle 6 with NEPA	CR Acute CINV	4	100
	CR Delayed CINV	4	100
Cycle 7 with NEPA	CR Acute CINV	1	100
	CR Delayed CINV	1	100
Cycle 8 with NEPA	CR Acute CINV	1	100
	CR Delayed CINV	1	100
Cycle 9 with NEPA	CR Acute CINV	1	100
	CR Delayed CINV	1	100

## Discussion

In this retrospective study, we evaluated the effectiveness of NEPA, a dexamethasone-sparing antiemetic regimen, for the prevention of CINV in cancer patients. A total of 69 cancer patients were included in the study, all of whom received NEPA in combination with single-dose dexamethasone (DEX) on day 1 of each chemotherapy cycle. The patient population consisted of individuals with various types of cancer, including head and neck, breast, lung, colorectal, and ovarian cancer. The primary outcome measure assessed was the complete response, i.e., the absence of vomiting or the need for rescue medication within 0-120 hours following chemotherapy administration, as reported by the patients to the principal investigator (PI). Analysis of the collected data revealed no reported cases of acute or delayed nausea and vomiting among the 69 cancer patients without requiring any rescue medication for CINV management. These findings indicate a relatively low incidence of the above-mentioned symptoms among the patients included in the study. Importantly, NEPA administration in cancer patients with cardiac comorbidities has not shown any significant cardiac or QTc prolongation effects, as observed in patients receiving 5-HT3 receptor antagonists which indicates that NEPA may be a safe option for preventing nausea and vomiting in this patient population. Overall, these results suggest that the dexamethasone-sparing antiemetic regimen utilizing NEPA demonstrated effectiveness in preventing CINV in the studied population. The absence of acute and delayed nausea and vomiting, along with no requirement of rescue medication, further supports the favorable effective profile of this treatment approach [8,13].

The results of the present study also demonstrated significantly improved complete response with the one-day DEX sparing antiemetic prophylaxis regimen using NEPA in comparison to aprepitant (APR) based setting NEPA [73.8% versus APR/GRAN 72.4%], which validates that NEPA is an effective option for the prevention of CINV in cancer patients. Importantly, the current antiemetic regimen had a dexamethasone-sparing approach, as none of the patients in our study received dexamethasone as part of their antiemetic treatment beyond day 1 of each chemotherapy cycle. This is particularly relevant considering the adverse effects associated with dexamethasone use, and the potential benefits of reducing its administration in cancer patients. Navari et al. conducted a real-world study among two cohorts of cancer patients who received cisplatin-based chemotherapy and the other arm received NEPA or fosaprepitant + palonosetron (APPA). The study regimens included palonosetron, but NK1RA components differed in each fosnetupitant and fosaprepitant. The results showed a reduced rate of nausea and vomiting in the NEPA group compared to APPA during all time periods post-chemotherapy, including 1-7 days following HEC. The results of which are comparable to the present study [14].

A posthoc pooled analysis of phase 3 study conducted by Aapro et al. demonstrated that NEPA when received orally had significant superiority in nausea control during the delayed and overall periods compared with palonosetron in the cisplatin and Doxorubicin and Cyclophosphamide (AC) based settings and a considerable advantage compared with aprepitant-based regimens in non-AC-based HEC [15]. Three pivotal, multicentre, randomized, double-blind and registration studies focusing on cisplatin-based highly HEC, who either received a single oral dose of NEPA plus dexamethasone before chemotherapy or aprepitant-based regimen (APR), showed delayed phase had notable differences in response rates between patients treated with NEPA plus dexamethasone and those receiving APR, 5-HT3RA, and dexamethasone. Specifically, 81.8% of patients on NEPA achieved complete responses compared to 76.9% of patients on APR. The improved outcomes observed with NEPA plus dexamethasone may be attributed to its longer half-life (80 hours) compared to that of aprepitant (9-13 hours) [16].

The trials conducted by Zhang et al. [17] and Aapro et al. [15] have shown that NEPA provides effective control of both acute and delayed CINV, making it suitable for patients receiving highly emetogenic chemotherapy regimens. The data pooled from a randomized, multicentred, pivotal cisplatin-based HEC phase 3 study conducted by Navari et al. to evaluate the effectiveness of single-dose Netupitant versus 3-day aprepitant for prevention of CINV, demonstrated superior effectiveness of NEPA compared to aprepitant regimen during 1-5 days following chemotherapy [16]. The efficacy of NEPA in the present study aligns with findings from these clinical trials which favour the implementation of the regimen in patients receiving HEC.

A study conducted by Chang et al. showed that >85% of control in nausea was observed when patients receiving high-dose chemotherapy were administered NEPA as compared to patients who received aprepitant and granisetron (APR/GRAN) which was 81% [10]. Escobar et al. in their study showed that nausea was seen in an increased number of patients (21.7%) while vomiting was observed in 9-16% of patients. These findings are in line with our study where eight patients experienced nausea while only three patients experienced vomiting [18]. The promotion of NEPA is advocated, given that the prevention of nausea represents the most significant gap in meeting the objective of mitigating CINV in individuals receiving emetogenic chemotherapy [10].

While conducting the present study, we acknowledge certain limitations that include the retrospective design and the absence of a control group. Retrospective studies, although valuable, may be influenced by inherent biases and potential confounding variables that can affect the results. To ensure the safety and validity of the study results, individuals who may have confounding factors or potential contraindications for the study intervention were excluded. Furthermore, using medical records as a source of data collection introduces the possibility of incomplete or missing data. However, despite these limitations, our study provides valuable insights into the subject matter and contributes to the existing body of knowledge in this field.

## Conclusions

The current retrospective study provides real-world evidence supporting the effectiveness of a dexamethasone-sparing antiemetic regimen with NEPA (Netupitant plus Palonosetron) for the prevention of chemotherapy-induced nausea and vomiting (CINV) in cancer patients. The study also demonstrated a high complete response rate for both acute and delayed CINV when NEPA was used as a one-day antiemetic prophylaxis regimen. The absence of vomiting and minimal rescue medication use within 24 hours after chemotherapy administration indicates the high efficiency of NEPA in reducing CINV. As we delve further into the use of NEPA, it is important to continue exploring its optimal utilization, potential drug interactions, and long-term safety data, ultimately improving CINV management and enhancing patients' treatment experience.
